# Fibrosis-4 (FIB-4) Index and mortality in COVID-19 patients admitted to the emergency department

**DOI:** 10.1007/s11739-022-02997-9

**Published:** 2022-05-27

**Authors:** Tommaso Bucci, Gioacchino Galardo, Orietta Gandini, Tommasa Vicario, Carla Paganelli, Sara Cerretti, Chiara Bucci, Francesco Pugliese, Daniele Pastori, the Research On Medical patients Admitted to the Emergency Department (ROMA-ED) study group, Elisa Fante, Fabrizio Urso, Enrico Baldini, Laura Zinnamosca, Maria Alessandroni, Grazia Loiudice, Chiara Boccardo, Enrico Petrillo, Giada Della Grotta, Leonardo Magrini, Marina Colzi, Cristiana Gianni, Federica Biamonte, Antonio Concistrè, Antonella Ponzio, Cristiana Franchi, Cristiano Marinelli, Tecla Pecci, Flavia Fabi, Giona Roma, Alessandra Massi, Alina Diaczenko, Emanuela Bresciani, Emanuela Bresciani, Marianna Suppa, Adriana Servello, Antonello Rosa, Alessandro Coppola, Anna Maria Mazzocchitti, Mariangela Palladino, Giuliano Bertazzoni, Salvatore Minisola, Luigi Petramala, Luca Marino, Andrea Marletta, Marco De Cataldis, Daniele Corbi, Rosachiara Ansalone, Adriana D’Ercole, Serena Fontana, Paolo Rapisarda, Pietro Piccari, Giulia Marcelli, Michela Cascio, Valentina Di Manno, Margherita Ruggiero, Giulia Cardillo Piccolino, Pierfrancesco Sinacori, Stefano Rossi, Domenico Di Vanna, Mauro Barbera, Maria Civita Cedrone, Valentina Di Biagio, Elisabetta Galati, Giulia Iacopelli, Annalisa Leonardi, Daria Rigamonti, Marco Colantonio, Annalisa Leonardi, Eugenia Pellegrino, Maria Antonietta Colafati, Mimosa Milocco, Rosaria Berardi, Danilo Menichelli, Giovanni Franchino, Anna Criniti, Carla Lubrano, Maria Santulli, Antonio Angeloni, Emiliano Lorusso, Simona Giglio

**Affiliations:** 1 Department of General Surgery and Surgical Specialties “Paride Stefanini”, Sapienza University of Rome, Rome, Italy; 2 Department of Molecular Medicine, Sapienza University of Rome, Rome, Italy; 3 Emergency Department, Policlinico Tor Vergata Hospital, Rome, Italy; 4 Emergency Medicine Unit, Department of Clinical Internal, Anesthesiological and Cardiovascular Sciences, Sapienza University of Rome, Rome, Italy

**Keywords:** COVID-19, FIB-4, Liver fibrosis, AST, ALT

## Abstract

Liver damage worsens the prognosis of coronavirus 19 disease (COVID-19). However, the best strategy to stratify mortality risk according to liver damage has not been established. The aim of this study is to test the predictive value of the validated Fibrosis-4 (FIB-4) Index and compared it to liver transaminases and to the AST-to-Platelet ratio index (APRI). Multicenter cohort study including 992 consecutive COVID-19 patients admitted to the Emergency Department. FIB-4 > 3.25 and APRI > 0.7 were used to define liver damage. Multivariable Cox regression and ROC curve analysis for mortality were performed. Secondary endpoints were (1) need for high-flow oxygen and (2) mechanical ventilation. 240 (24.2%) patients had a FIB-4 > 3.25. FIB-4 > 3.25 associated with an increased mortality (*n* = 119, log-rank test *p* < 0.001 and adjusted hazard ratio (HR) 1.72 (95% confidence interval [95%CI] 1.14–2.59, *p* = 0.010). ROC analysis for mortality showed that FIB-4 (AUC 0.734, 95% CI 0.705–0.761) had a higher predictive value than AST (*p* = 0.0018) and ALT (*p* < 0.0001). FIB-4 > 3.25 was also superior to APRI > 0.7 (AUC 0.58, 95% CI 0.553–0.615, *p* = 0.0008). Using an optimized cut-off > 2.76 (AUC 0.689, 95% CI 0.659–0.718, *p* < 0.0001), FIB-4 was superior to FIB-4 > 3.25 (*p* = 0.0302), APRI > 0.7 (*p* < 0.0001), AST > 51 (*p* = 0.0119) and ALT > 42 (*p* < 0.0001). FIB-4 was also associated with high-flow oxygen use (*n* = 255, HR 1.69, 95% CI 1.25–2.28, *p* = 0.001) and mechanical ventilation (*n* = 39, HR 2.07, 95% CI 1.03–4.19, *p* = 0.043). FIB-4 score predicts mortality better than liver transaminases and APRI score. FIB-4 score may be an easy tool to identify COVID-19 patients at worse prognosis in the emergency department.

**Supplementary Information:**

The online version contains supplementary material available at 10.1007/s11739-022-02997-9.

## Introduction

Severe acute respiratory syndrome coronavirus 2 (SARS-CoV-2) infection may cause a systemic inflammatory disease causing not only acute respiratory failure but also multi-organ damage. This is the consequence of the ubiquitous distribution of the angiotensin converting enzyme 2 (ACE2) [1], and to the systemic release of pro-inflammatory [2] and pro-thrombotic compounds [3]. Indeed, there have been described several cases of cardiac, renal and liver involvement during the coronavirus 19 disease (COVID-19) [4].

In particular, the presence of liver damage seems to be quite common in COVID-19 patients with an estimated prevalence of patients with elevated aspartate aminotransferase (AST) of 23.2% and alanine aminotransferase (ALT) of 21.2% [5]. In addition, several evidence suggested that liver injury is associated with a more severe SARS-CoV-2 infection [6–8], especially when liver damage was defined by raised AST [5]. The presence of liver damage was also shown to represent a negative prognostic factor for COVID-19 patients [9, 10]. However, previous studies on COVID-19 patients mostly used liver transaminases to define liver damage and to describe its association with mortality risk [9].

However, it has become evident that non-invasive scores may identify patients with liver impairment better than liver transaminases. In this context, the Fibrosis-4 (FIB-4) Index and AST-to-Platelet ratio index (APRI) are the two most widely investigated scores that showed a good correlation with the presence of liver fibrosis detected at liver biopsy in different clinical settings, including viral hepatitis, alcoholic and non-alcoholic fatty liver disease[11]. The advantage of these non-invasive scores is to detect relevant liver damage also in patients with nearly normal or only mild elevation of liver transaminases and may save a significant number of unnecessary liver biopsy procedures.

In addition, previous studies showed that these non-invasive scores may have a prognostic role for cardiovascular events and mortality both in liver [12] and non-liver diseases [13].

Scarce data on the COVID-19 population have been reported so far; one previous study showed that FIB-4 was associated with the need for mechanical ventilation but no data on mortality were reported [14]. Conversely, studies on mortality risk according to FIB-4 included a relatively small sample or specific subgroups of patients, such as those with liver steatosis [15, 16] or haematological malignancies [17], and used different cut-off of FIB-4, making results of difficult comparison [18]. Given the still wide-spread diffusion of SARS-CoV-2 infection, more data allowing a better risk stratification strategy and eventually sources allocation, are warranted.

To this aim, we compared the prognostic value of liver transaminases, FIB-4 and APRI score with mortality risk in a large population of consecutive COVID-19 patients admitted to the Emergency Department of two University Hospitals in Rome.

## Patients and methods

We carried out a retrospective multicenter cohort study including on 992 patients, affected by COVID-19, admitted to the Emergency Department of Umberto I University Hospital in Rome and from Tor Vergata University Hospital of Rome from March to October 2020. All patients were diagnosed with COVID-19 after two positive polymerase chain reaction tests on nasopharyngeal swab specimens. Patients aged < 18 years were excluded, as well as patients with history of liver cirrhosis. To limit bias, no additional exclusion criteria were applied.

The following data were collected from at the time of COVID-19 diagnosis in the emergency department: demographic, comorbidities, clinical, laboratory and radiological findings. Patients underwent a routine laboratory screening at the entry of the Emergency Department including, complete blood count (CBC), lactate dehydrogenase (LDH), C-reactive protein (CRP), ferritin, D-dimer, creatinine with estimated Glomerular Filtration Rate (eGFR) estimation (MDRD formula), Aspartate aminotransferase (AST), Alanine aminotransferase (ALT), GGT. Chronic kidney disease (CKD) was defined by and eGFR < 60 ml/min. An arterial blood gas analysis was also obtained and the corresponding PaO_2_/FiO_2_ ratio evaluated.

### Radiological findings

All patients underwent high-resolution chest computed tomography (CT) to evaluate the presence of interstitial pneumonia and its severity. Patients were classified as (1) no pneumonia if there was no radiological sign of pneumonia, (2) mild pneumoniae if there was only interstitial involvement without consolidation, (3) moderate pneumoniae if there was interstitial involvement with consolidation in less of 50% of lung parenchyma and (4) severe pneumoniae if there was interstitial involvement and consolidation in more than 50% of lung parenchyma.

### Non-invasive scores

FIB-4 score was calculated as follows: age year × AST (U/L)/Platelet Count (1000∕L) × √ALT (U/L). A cut-off of > 3.25 was used to define liver damage. For the analysis, a specific cut-off of > 2.76 for the study cohort was obtained from ROC analysis. As a second marker we calculated the APRI score as follows: [(AST/upper limit of the normal AST range) × 100]/Platelet Count. A cut-off of > 0.70 was used to define liver damage.

### Follow-up and mortality

After the initial evaluation and management, patients were discharged in home isolation or were hospitalized in low, medium or sub-intensive/intensive care units according to medical needs. All patients were followed up to 60 days after the Emergency Department admission. The principal endpoint of the study was all-cause mortality. Deaths were double checked on electronic records.

In keeping with statements by the Italian Regulatory Authorities (https://www.garan tepri vacy.it/web/guest/ home/docwe b/-/docweb-display/docwe b/5805552), anonymised data were retrospectively collected from medical and electronic databases in the context of an audit. Patients were not directly involved in any phase of the study. A waiver of informed consent from study participants is applied for retrospective studies. The study was conducted in accordance with the principles embodied in the Declaration of Helsinki.

## Statistical analysis

Continuous variables are reported as mean and standard deviation or median and interquartile range depending on variable distribution. Means and medians were compared by Student’s *t* test or the Mann–Whitney *U* test, respectively. Categorical variables were reported as count and percentage and compared by Pearson chi-squared test. A first descriptive analysis of clinical, biochemical and radiological characteristics of patients was performed according to the presence of FIB-4 above or below 3.25.

We then analysed factors associated with mortality risk using univariable and multivariable Cox proportional hazard regression analysis with forward stepwise selection procedure. For the analysis, linear variables were categorised into tertiles. Only variables with complete data available were used for the multivariable model.

We also built the receiver operating characteristic (ROC) curves to test the predictive value of FIB-4, APRI, AST and ALT against in-hospital mortality. Area under the curve (AUC) values were calculated using the method described by Delong et al. [19]. In addition, we used the ROC curve with Youden index to find the optimal cut off for FIB-4 (> 2.76), AST (> 51) and ALT (> 42) against mortality. Secondary endpoints were the need for high flow oxygen, such as non-invasive ventilation (NIV) or high flow nasal cannula (HFNC) and mechanical ventilation. Multivariable models for secondary endpoints were adjusted for the same variables as for the primary one. The statistical significance was set at a *p* value < 0.005. All the analyses were performed using the IBM software SPSS 25.0 and MedCalc^®^.

## Results

### Clinical characteristics

The demographics and clinical characteristics of COVID-19 patients according to the FIB-4 score are shown in Table 1. In the whole cohort, 240 had a FIB-4 > 3.25 (24.2%). Patients with FIB-4 > 3.25 were older and showed a higher prevalence of hypertension, diabetes, heart failure and active cancer. Regarding the clinical presentation, they presented more frequently fever, low peripheral oxygen saturation, signs of severe respiratory failure (PaO_2_/FiO_2_ ratio < 200) and extensive pneumonia at the high-resolution chest CT (Table 1). Amongst the laboratory variables, patients with FIB-4 > 3.25 had a lower median eGFR, lymphocytes and platelet count and a higher median concentration of D-dimer, serum ferritin, CRP and LDH. At baseline, patients with high FIB-4 were taking a higher number of cardiovascular drugs (Table 1). Regarding the COVID-19 treatment modalities, no differences were noted about anticoagulation and steroids prescription.

**Table 1:** Comparison of patients with SARS-CoV-2 infection based on FIB-4 score

Variables	Total population *n* = 992% (*n*)	FIB-4 < 3.25 *n* = 752% (*n*)	FIB-4 > 3.25 *n* = 240% (*n*)	*p*
Age (years)	61 (54–70)	57 (51–64)	76 (70–81)	< 0.001
Women	39.6 (393)	41.2 (310)	34.6 (83)	0.067
Arterial hypertension	34.3 (341)	30.6 (230)	46.2 (111)	< 0.001
Diabetes	19.1 (189)	16.2 (122)	28.6 (67)	< 0.001
Heart failure (*N* = 641)	9.4 (60)	7.6 (36)	14.5 (24)	0.013
COPD (*N* = 919)	9.1 (84)	8.3 (58)	11.8 (26)	0.114
Cancer	5.7 (57)	4.5 (34)	9.6 (23)	0.003
Concomitant treatments^a^				
Proton pump inhibitor	16.9 (90)	12.8 (52)	29.5 (38)	< 0.001
ACE inhibitors	15.0 (80)	13.1 (53)	20.8 (27)	0.047
Sartans	11.6 (62)	10.9 (44)	13.8 (18)	0.348
Diuretics	6.9 (37)	4.7 (19)	13.8 (18)	0.001
Statins	11.8 (63)	8.9 (36)	20.8 (27)	0.001
Calcium channel blockers	8.8 (47)	7.9 (32)	11.5 (15)	0.214
Beta-blockers	13.1 (70)	11.1 (45)	19.2 (25)	0.024
Antiplatelet	15.0 (80)	10.6 (43)	28.5 (37)	< 0.001
Insulin	4.2 (15)	3.1 (9)	9.0 (6)	0.043
Vital signs				
Heart rate	88 (80–94)	88 (80–94)	88 (80–94)	0.727
Systolic blood pressure	130 (120.130)	130 (120–130)	130 (120–130)	0.448
Diastolic blood pressure	70 (70–80)	70 (70–80)	70 (70–80)	0.224
O_2_ saturation	97 (96–98)	97 (96–98)	95.5 (93–97)	< 0.001
Temperature (°C)	37 (36.5–37.5)	37 (36.5–37.5)	37.3 (36.8–38)	0.001
pO_2_	74 (66–98)	77 (69–86)	66 (56–75)	< 0.001
PaO_2_/FiO_2_	295 (251–388)	357 (266–400)	258 (210–291)	< 0.001
PaO_2_/FiO_2_ < 200	18 (179)	13.6 (102)	32.1 (77)	< 0.001
Radiological findings				
No pneumonia	9 (89)	10.8 (81)	3.3 (8)	< 0.001
Mild pneumonia	23.3 (231)	25.3 (190)	17.1 (25.3)	
Moderate pneumonia	42.4 (421)	40.8 (307)	47.5 (114)	
Severe pneumonia	25.4 (252)	23.1 (174)	32.1 (77)	
Laboratory findings				
White blood cell (× 10^3^/µL)	5.9 (5–7.1)	6.03 (5.1–7.3)	5.3 (4.2–6.8)	< 0.001
Neutrophils (× 10^3^/µL)	3.9 (3–5.1)	4.1 (3.1–5.2)	3.7 (2.5–4.8)	0.004
Lymphocytes (× 10^3^/µL)	0.9 (0.6–1.1)	0.9 (0.7–1.2)	0.7 (0.5–0.9)	< 0.001
Platelets (× 10^3^/µL)	187 (162–220)	207 (178–236)	142.5 (120.3–158.6)	< 0.001
Platelets < 150 (× 10^3^/µL)	24.2 (240)	13.3 (100)	58.3 (140)	< 0.001
D-dimer (ng/mL) (*N* = 762)	664 (464–937)	606 (431–816)	973 (606–1456)	< 0.001
Ferritin (ng/mL) (*N* = 453)	595 (383–914)	535 (354–841)	829 (536–1339)	< 0.001
C-reactive protein (mg/dL) (*n* = 766)	3.7 (1.8–6.3)	3.2 (1.3–5.6)	6 (3.7–9.2)	< 0.001
LDH (U/L) (*n* = 865)	289 (228.5–387.5)	282.5 (223–371)	401 (260.5–560.5)	< 0.001
Alanine aminotransferase (U/L)	24 (18–32)	25 (18–34)	22 (16–30)	0.020
Aspartate aminotransferase (U/L)	31 (25–38)	28 (23–35)	40 (33–49)	< 0.001
GGT (U/L) (*N* = 584)	28 (20–41)	28 (21–39.3)	26 (18–44)	0.683
eGFR (mL/min)	81 (70–92.4)	87.1 (74.9–96)	65 (51.3–77.5)	< 0.001

^a^Data available in 535 patients (405 with FIB-4 < 3.25 and 130 with FIB-4 > 3.25)

## Primary outcome

**Fig. 1:**
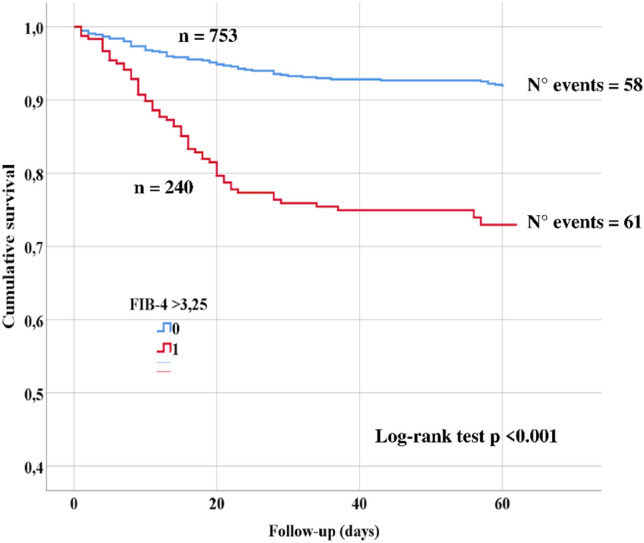
Kaplan–Meier curves of survival of patients according to FIB-4 values

After the initial evaluation, patients with FIB-4 < 3.25 were more frequently discharged at home or hospitalized in low-intensity care units while patients with FIB-4 > 3.25 were more often hospitalized in sub-intensive and intensive care units (Supplementary table 1). In our population of COVID-19, the mean follow-up was 50 ± 18 days. During follow-up 119 deaths (13%) were recorded. The prevalence of FIB-4 > 3.25 was higher in non-survivor vs survivor patients (51.3% vs. 20.5%; *p* < 0.0001). Patients with FIB-4 > 3.25 showed a higher incidence of death than patients with FIB-4 < 3.25 (7.6% vs. 25.4%; log-rank test *p* < 0.001; Fig. 1). At univariable regression analysis (Supplementary Table 1), factors associated with mortality were age > 70 years. hypertension, diabetes, heart failure, COPD, cancer, CKD, PaO_2_/FiO_2_ < 200, CT signs of severe pneumoniae, lymphocytes < 0.6 × 10^3^/µL, high D-dimer > 937 ng/mL, serum ferritin > 914 ng/mL, CRP > 6.3 mg/dL, LDH, eGFR, AST, ALT and FIB-4 > 3.25 (Supplementary Table 1). In the multivariable regression model, CKD, PaO_2_/FiO_2_ < 200, CRP > 6.3 mg/dL and FIB-4 > 3.25 were independently associated with mortality (Table 2).

**Table 2:** Multivariable Cox proportional hazards regression analysis for mortality (A), HFNC/NIV (B), mechanical ventilation (C)

Mortality	Hazard ratio	95% Confidence interval	p
FIB-4 > 3.25	1.72	1.14–2.59	0.010
Age > 70 years	2.92	1.81–4.72	< 0.001
Female sex	0.88	0.60–1.31	0.533
Diabetes	1.51	1.03–2.23	0.036
Arterial hypertension	2.02	1.35–3.00	0.001
Cancer	1.80	1.02–3.18	0.043
PaO_2_/FiO_2_ < 200	3.68	2.53–5.36	< 0.001
Lymphocytes < 0.6 (× 10^3^/µL)	0.91	0.61–1.34	0.628
HFNC/NIV			
FIB-4 > 3.25	1.69	1.25–2.28	0.001
Age > 70 years	1.10	0.81–1.49	0.555
Female sex	0.81	0.62–1.07	0.144
Diabetes	1.21	0.91–1.62	0.188
Arterial hypertension	1.66	1.27–2.18	< 0.001
Cancer	1.69	1.05–2.73	0.030
PaO_2_/FiO_2_ < 200	9.91	7.52–13.06	< 0.001
Lymphocytes < 0.6 (× 10^3^/µL)	0.85	0.65–1.10	0.221
Mechanical ventilation			
FIB-4 > 3.25	2.07	1.03–4.19	0.043
Age > 70 years	2.65	1.29–5.46	0.008

## ROC analysis

**Fig. 2:**
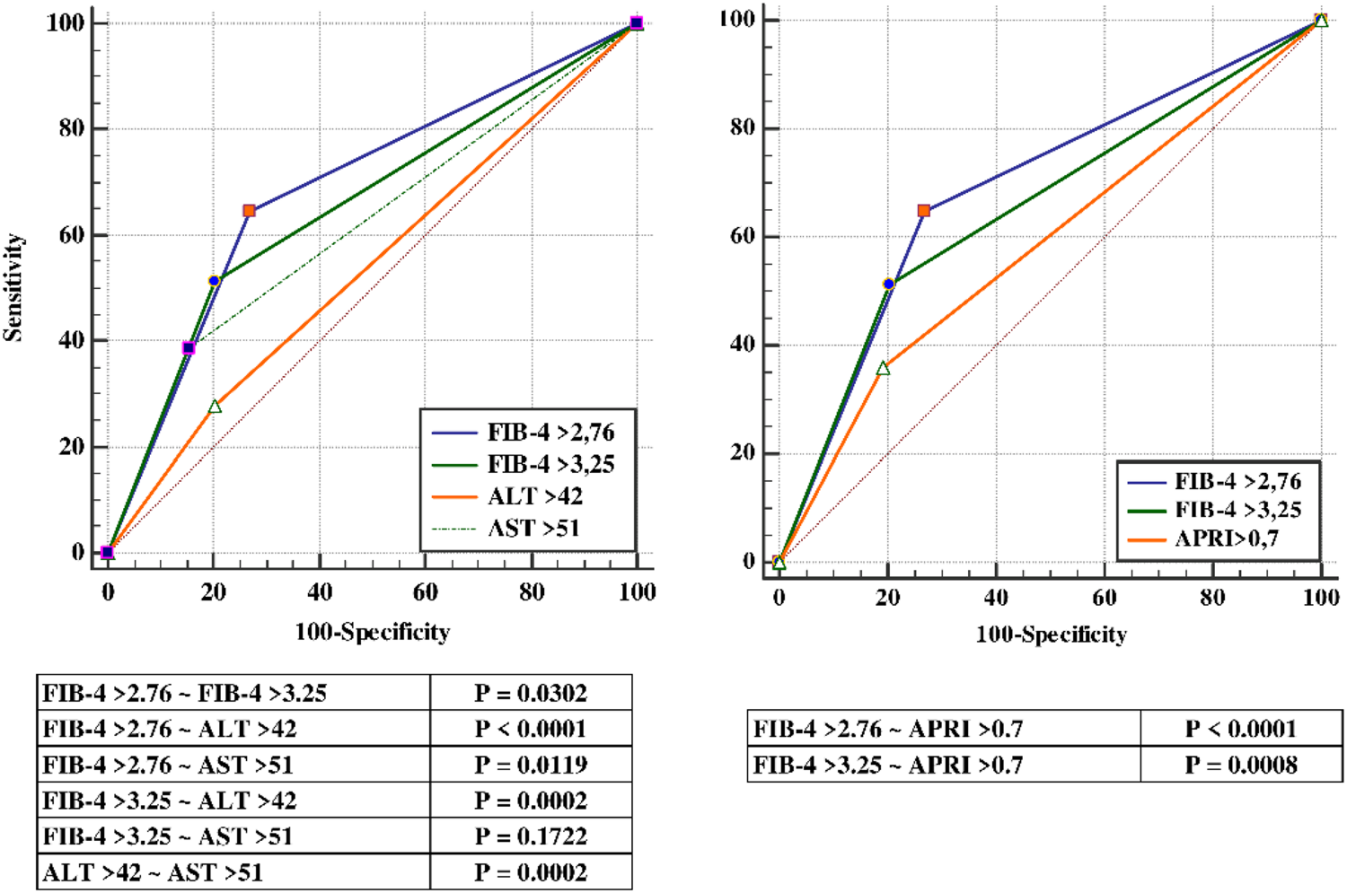
Receiver operating characteristic (ROC) curves of FIB-4 score against mortality compared to categorized AST, ALT and APRI score

At ROC analysis (Table 3), FIB-4 score, as a continuous variable, showed a higher predictive value than AST and ALT (AUC 0.73, 0.64 and 0.51, respectively, Supplementary Fig. 1). FIB-4 > 3.25 was superior to ALT and APRI > 0.7 in predicting mortality (Fig. 2). In particular, we found that the optimal cut-off of > 2.76, obtained from the ROC analysis, was superior to FIB-4 > 3.25, APRI > 0.7 and both ALT and AST even when optimal cut-offs for these variables were used (Fig. 2).

**Table 3:** ROC curve analysis

Variables	AUC	95% Confidence interval	*p* value
FIB-4 (continuous)	0.73	0.71–0.76	< 0.0001
FIB-4 > 3.25	0.66	0.62–0.68	< 0.0001
FIB-4 > 2.76	0.69	0.66–0.72	< 0.0001
APRI (continuous)	0.64	0.61–0.67	< 0.0001
APRI > 0.7	0.58	0.55–0.62	0.0003
AST (continuous)	0.64	0.61–0.67	< 0.0001
AST > 51	0.62	0.59–0.65	< 0.0001
ALT (continuous)	0.51	0.48–0.54	0.8176
ALT > 42	0.54	0.51–0.57	0.0897

## Secondary outcomes

Patients with FIB-4 > 3.25 require more often oxygenation with HFNC, NIV or mechanical ventilation (Supplementary Table 2).

Overall, 255 (25.7%) patients were treated with HFNC/NIV, 20.5% of survivors vs. 69.7% of non-survivors (*p* < 0.001). Univariable HR for FIB-4 > 3.25 for HFNC/NIV was 2.72, 95% CI 2.12–3.49, *p* < 0.001. FIB-4 > 3.25 remained associated with an increased risk for HFNC/NIV in the multivariable model (HR 1.69, 95% CI 1.25–2.28, *p* = 0.001. Supplementary Table 2).

Thirty-nine (3.9%) patients underwent mechanical ventilation, 1.9% of survivors and 18.5% of non-survivors. Univariable HR for FIB-4 > 3.25 for mechanical ventilation was 3.24, 95% CI 1.72–6.08, *p* < 0.001. FIB-4 > 3.25 remained associated with an increased risk for mechanical ventilation in the multivariable model (HR 2.07, 95% CI 1.03–4.19, *p* = 0.043. Table 3).

## Discussion

In this multicenter cohort study, we found that the FIB-4 may be an easy and accurate tool to predict mortality in patients with COVID-19. We showed that the FIB-4 was superior to liver transaminases alone or to APRI score to predict mortality, especially when a COVID-19 adapted cut-off of FIB-4 was tested.

A first meaningful result is that nearly 25% of patients hospitalized with COVID-19 showed a high FIB-4 > 3.25 and 31.4% for FIB-4 > 2.76, suggesting that a high proportion of patients may have an early liver involvement during COVID-19. This figure is similar to that reported in a smaller cohort of 202 COVID-19 patients in which the prevalence of FIB-4 > 2.67 was 31.2% [18]. In addition, in a study using a FIB-4 cut off > 2.91 the prevalence of high FIB-4 was 24.9% [15].

Patients with a high FIB-4 were older, with a high prevalence of comorbidities including arterial hypertension and diabetes. Furthermore, FIB-4 patients had a more severe clinical presentation of COVID-19 as shown by a lower pO_2_ and PaO_2_/FiO_2_. They also showed a pro-inflammatory and pro-thrombotic phenotype as shown by increased D-Dimer, CRP, Ferritin, all features associated with severe respiratory failure [20–22].

When we analyzed clinical outcomes, we found a mortality rate of 13% within 60 days from the admission to the emergency department. This finding is slightly higher than the 10.9% reported in the study by Li Y. et al. [18] and 10.8% in the study by Younossi et al. [15]. Of note, we found a higher prevalence of increased FIB-4 in patients who died compared to survivors. This association persisted in the multivariable survival model after adjustment for potential confounders and for the severity of respiratory failure. In particular, when we built the ROC curves for mortality, we found an AUC of 0.73 for the FIB-4 score that is in line with recent studies [23, 24].

A novel finding of this work relies on the comparison of the prognostic role of the FIB-4 score for mortality with liver transaminases and with another commonly used score such as the APRI score. We firstly examined the cut-off of 3.25 as it is the most widely used in previous studies, and we found that it was significantly associated with mortality. Furthermore, it allowed a better prediction of mortality than ALT and APRI > 0.7. Then from ROC curve analysis, we found that in this patients’ population, a value > 2.76 showed the best combination of sensitivity and specificity. We repeated survival analysis using this optimized cut-off and found an improvement in risk prediction compared to the 3.25 value and to AST, ALT and APRI also using optimized cut-offs for these variables. The predictive value for mortality of the FIB-4 score seems also to be higher than other previously tested scores for mortality in COVID-19 patients, namely WHO severity scale, NEWS, CURB-65 and APACHE scores (all AUC values < 0.66) [25].

We also analyzed the association between a high FIB-4 and indexes of severe COVID-19, such as the need for high oxygen flow and mechanical ventilation. We found that FIB-4 patients had a nearly doubled risk of being treated with high oxygen flow or of needing mechanical ventilation. This association was similar to that reported in a recent study, which reported a 6% of mechanical ventilation and using a cut-off for high FIB-4 set at 3.04 [14].

Our results suggest that liver damage, when evaluated by the FIB-4 score, may be a risk factor for mortality independently from the severity of COVID-19. Indeed, FIB-4, that is calculated using routine laboratory variables, may be an easy prognostic tool to stratify mortality risk in COVID-19 patients admitted to the emergency department.

Limitations and strengths. Our study evaluated the presence of liver damage only at admission, so we do not know whether in some cases liver damage was worsened by concomitant treatments during the hospital stay or if it persisted after the acute phase of COVID-19. We do not have data on viral infections as they are not routinely tested in the emergency department. Our cohort is composed by Caucasian patients only and, therefore, our findings may not apply to other ethnic groups. The retrospective nature of the study does not allow to establish any cause–effect relationship. However, we analysed a quite large cohort of consecutive unselected patients referring to the emergency unit, so that our cohort is representative of patients encountered in daily clinical practice. Furthermore, the cohort is well characterized as all patients underwent CT chest scan, blood gas analysis and an accurate medical personal history collection. Finally, data were collected from medical records and not from ICD codes.

In conclusion, FIB-4 score showed a good predictive value for mortality in patients admitted to the Emergency Department for COVID-19. Its use may help physicians to early identify patients at higher risk for a more severe disease and at higher risk of mortality.

## Supplementary Information

Below is the link to the electronic supplementary material.

Supplementary file1 (DOCX 213 KB)
