# Supplementary material for: Fibrosis-4 (FIB-4) Index and mortality in COVID-19 patients admitted to the emergency department
Source: Intern Emerg Med. 2022 May 27;17(6):1777–84. doi: 10.1007/s11739-022-02997-9 (PMC9140323; doi:10.1007/s11739-022-02997-9)
Supplement: Supplementary file 1 — Supplementary file1 (DOCX 213 KB) [file 11739_2022_2997_MOESM1_ESM.docx]

**Fibrosis-4 (FIB-4) Index and mortality in COVID-19 patients admitted to the Emergency Department**

**Supplementary data**

**Supplementary table 1. Univariable Cox proportional hazards regression analysis for mortality.**

|  | Hazard Ratio | 95% Confidence Interval | p |
| --- | --- | --- | --- |
| Age >70 years (highest tertile) | 5.67 | 3.78-8.50 | < 0.001 |
| Female sex | 0.80 | 0.55-1.16 | 0.237 |
| Arterial hypertension | 3.38 | 2.34-4.89 | < 0.001 |
| Diabetes | 2.41 | 1.65-3.52 | < 0.001 |
| Heart Failure | 3.70 | 2.30-5.95 | < 0.001 |
| COPD | 2.75 | 1.75-4.36 | < 0.001 |
| Cancer | 2.37 | 1.35-4.14 | 0.002 |
| eGFR <60 ml/min | 5.53 | 3.85-7.95 | < 0.001 |
| PaO_2_/FiO_2_ < 200 | 5.05 | 3.53-7.24 | < 0.001 |
| CT signs of severe pneumonia | 2.64 | 1.84-3.79 | < 0.001 |
| Lymphocytes < 0.6 (lowest tertile) | 1.53 | 1.05-2.24 | 0.026 |
| D-dimer > 937 (highest tertile) | 3.84 | 2.57-5.72 | < 0.001 |
| Ferritin > 914 (highest tertile) | 2.64 | 1.61-4.33 | < 0.001 |
| CRP > 6.3 (highest tertile) | 2.86 | 1.80-4.53 | < 0.001 |
| FIB-4 > 3.25 | 3.75 | 2.61-5.37 | < 0.001 |
| FIB-4 > 2.76 | 4.52 | 3.10-6.58 | < 0.001 |
| AST continuous | 1.004 | 1.002-1.006 | < 0.001 |
| AST > 51 U/L | 3.14 | 2.17-4.54 | < 0.001 |
| ALT continuous | 1.002 | 0.999-1.005 | 0.141 |
| ALT >42 U/L | 1.44 | 0.96-2.14 | 0.077 |
| APRI >0.7 | 2.24 | 1.54-3.25 | <0.001 |
| Platelet count | 1.000 | 0.99-1.00 | 0.985 |

*vs no pneumonia, mild and moderate

**Supplementary table 2. Ventilation and destination of patients according to FIB-4**

| Variables | Total population  n=992  % (n) | FIB-4 <3.25  n= 752  % (n) | FIB-4 >3.25  n= 240  % (n) | p |
| --- | --- | --- | --- | --- |
| Ventilation modalities and medication | | | | |
| Need for oxygen | 90 (857) | 87.5 (624) | 97.5 (232) | <0.001 |
| HFNC/NIV | 26.6 (264) | 20.8 (149) | 44.5 (106) | <0.001 |
| Invasive ventilation | 3.9 (39) | 2.8 (21) | 7.5 (18) | 0.001 |
| Steroids | 50 (486) | 49.1 (363) | 50.2 (118) | 0.649 |
| Anticoagulants | 46 (445) | 45.9 (338) | 45.5 (106) | 0.909 |
| Destination | | | | |
| Home discharge | 9.1 (90) | 11 (83) | 2.9 (7) | <0.001 |
| Low intensity care unit | 30.7 (304) | 32.3 (242) | 25.8 (62) |  |
| Moderate intensity care unit | 46.5 (461) | 45.5 (342) | 49.6 (119) |  |
| Sub intensive / intensive care unit | 13.7 (136) | 11.2 (84) | 21.7 (136) |  |

**Supplementary figure 1. Receiver operating characteristic (ROC) curves of FIB-4 score against mortality compared to AST and ALT continuous values**

**
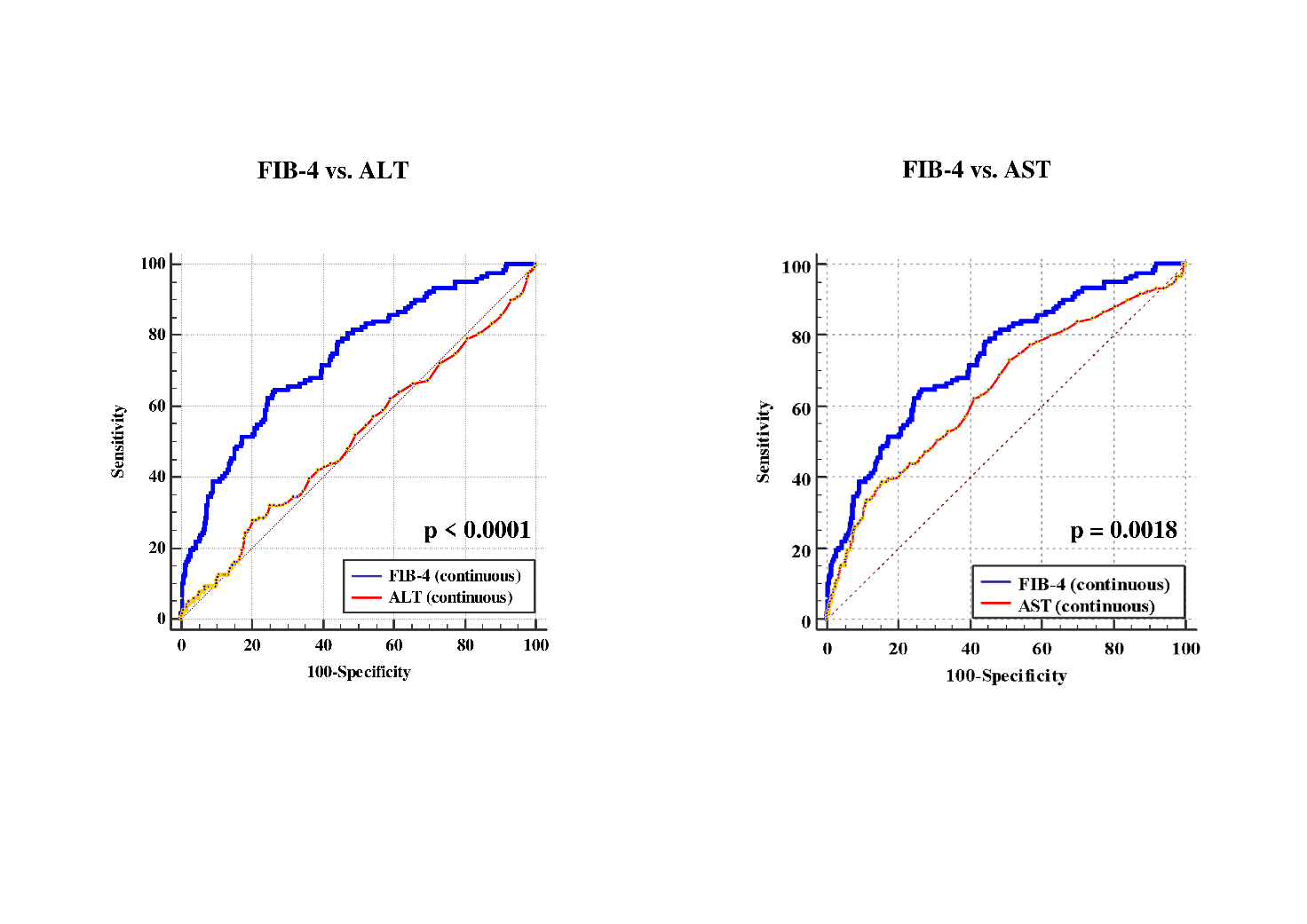
**
